# Supplementary material for: Centrosomal Protein 55 Regulates Chromosomal Instability in Cancer Cells by Controlling Microtubule Dynamics
Source: Cells. 2024 Aug 20;13(16):1382. doi: 10.3390/cells13161382 (PMC11353242; doi:10.3390/cells13161382)
Supplement: Supplementary file 1 [file cells-13-01382-s001.zip › cells-3140074-supplementary.pdf]

Centrosomal Protein 55 increases chromosomal instability in cancer cells by controlling microtubule dynamics

Stefanie Muhs[1], Themistoklis Paraschiakos[1], Paula Schäfer[1], Simon A. Joosse[2] and Sabine Windhorst[1]

[1]Department of Biochemistry and Signal Transduction, University Medical Center Hamburg-Eppendorf, Martinistrasse 52, D-20246 Hamburg

[2]Department of Tumor Biology, [2]Mildred Scheel Cancer Career Center HaTriCS4, University Medical Center Hamburg-Eppendorf, 20246 Hamburg, Germany.

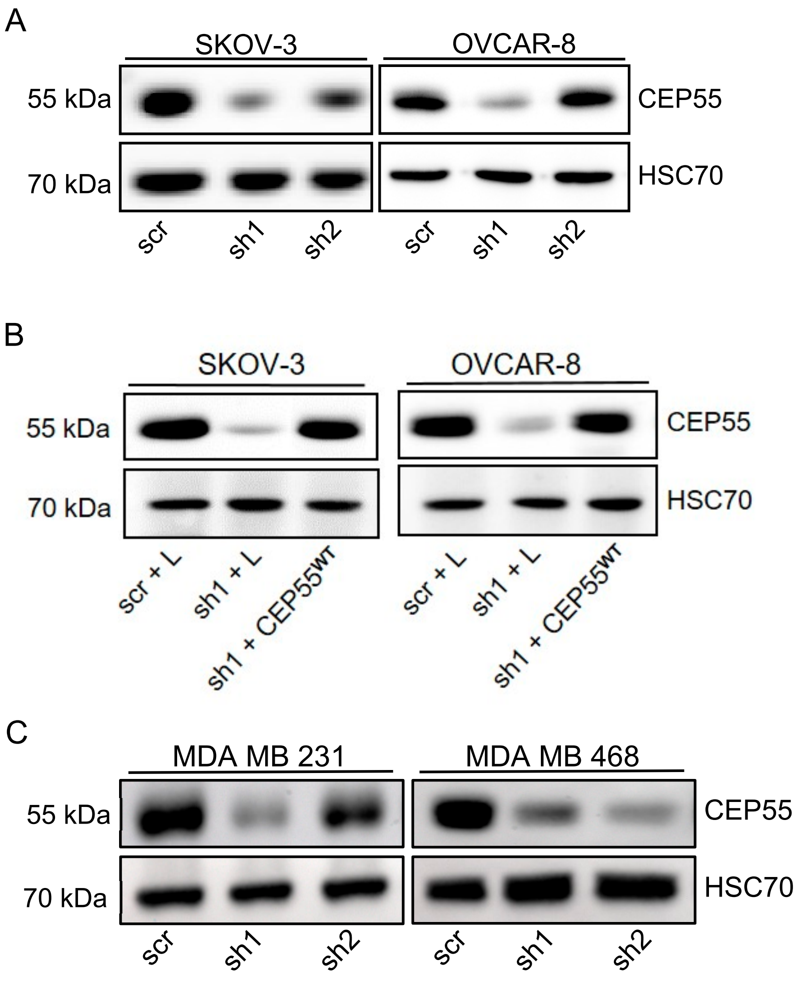

Figure S1. CEP55 manipulation in OvCa and breast cancer cells. (A) CEP55 was stably down-regulated by a lentiviral approach in ovarian cancer cell lines and the success of CEP55 knock-down was assessed by Western blotting. **(B)** CEP55 was stably re-expressed in knock-down cells using the lentiviral Lego (L) vector. Shown is one representative Western-blot analysis. **(C)** CEP55 was stably down-regulated by a lentiviral approach in breast cancer cell lines and the success of CEP55 knock-down was assessed by Western blotting.

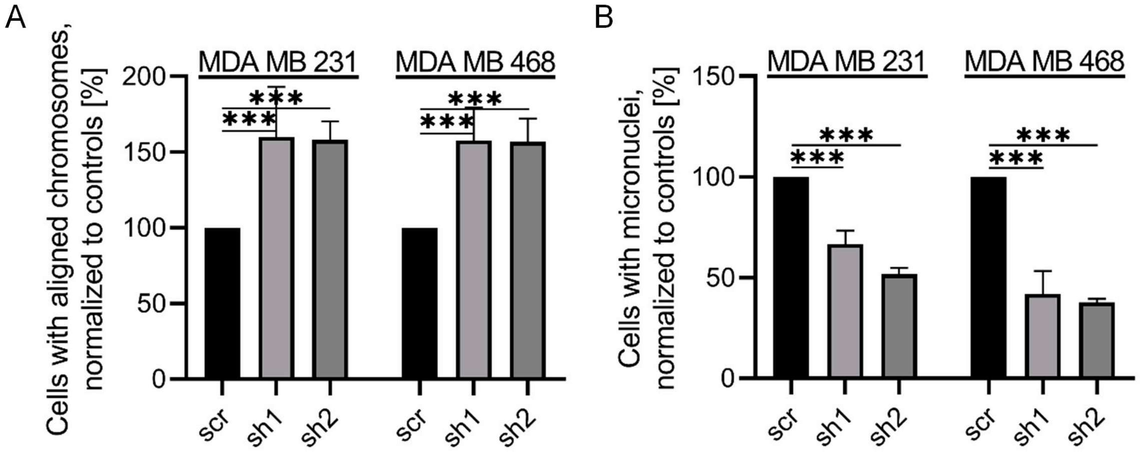

Figure S2. CEP55 depletion decreases the CIN rate in triple-negative breast cancer cells. (A) ß-tubulin and DAPI stained cells were grouped into cell populations with aligned or not aligned chromosomes and counted. The number of scrambled (scr) control cells with aligned chromosomes was set to 100%. **(B)** Interphase cells were stained using DAPI, the number of cells having micronuclei were counted and the percentage of cells with micronuclei was calculated. Significance was calculated by Chi^2 test. **p<0.001, ***p<0.0001.

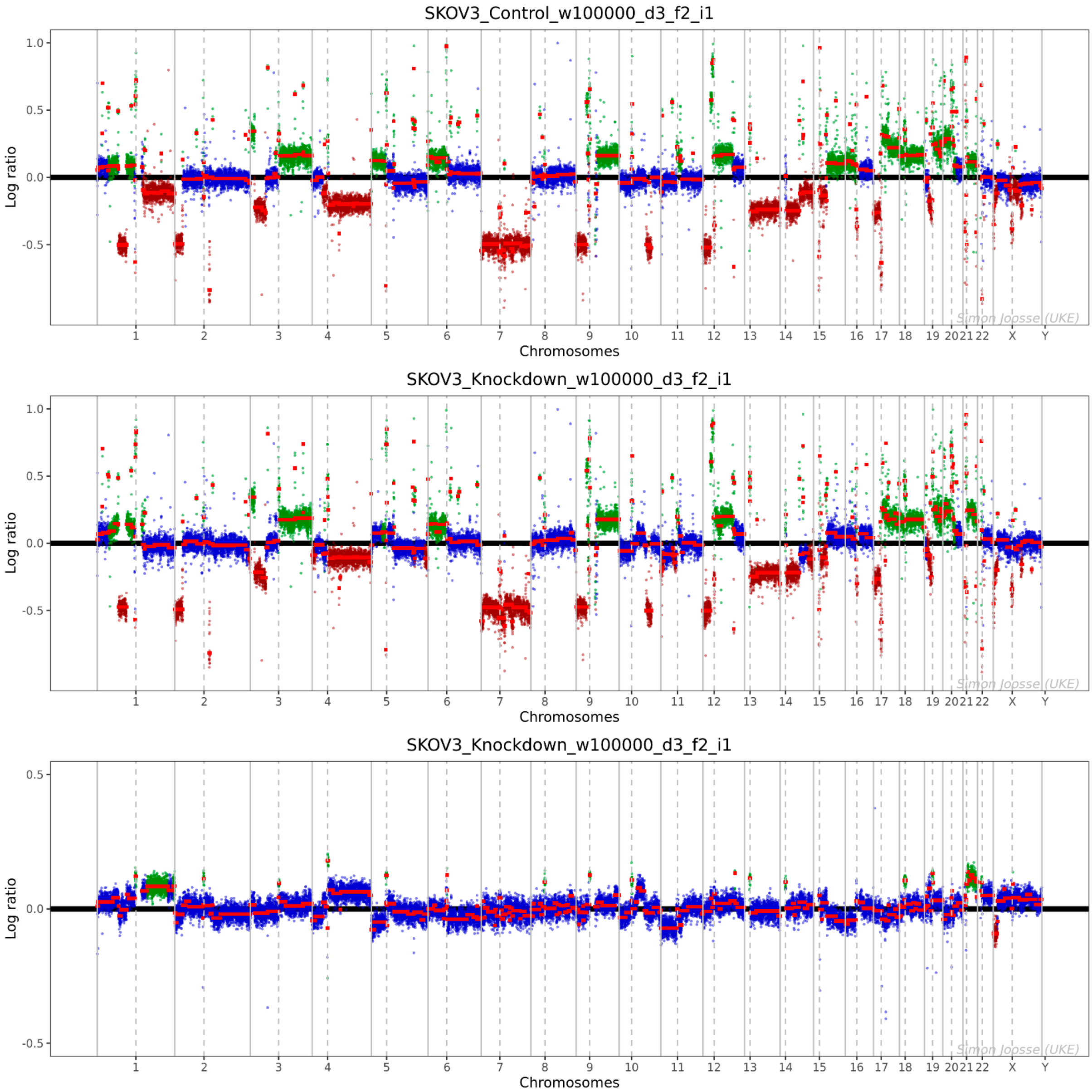

Figure S3. Copy number alterations (CNAs) in SKOV-3 cells. The **upper panel** depicts the CNAs of the control SKOV-3 cells, whereas the **middle panel** depicts the CNAs of the CEP55 knock-down cells. The chromosomes are depicted on the x-axis, with the solid lines signifying the chromosomal borders and the dotted lines the centromeres. The copy number levels are estimated based on the measure log ratio and copy number gains are depicted in green (positive log ratio), losses in red (negative log ratio), and no change in blue (log ratio around 0). The **bottom panel** depicts the difference between the control and knock-down cell lines. Log ratios corresponding to the same genomic loci were subtracted (knock-down – control). More extreme copy number alterations in the CEP55 knock-down cell lines as compared to

the control cell lines (knock-down$_{gain}$ > control$_{gain}$ | knock-down$_{loss}$ < control$_{loss}$), are described as “further away from baseline”, and are depicted as red shaded regions. However, if the copy number alterations were more extreme (i.e., further away from baseline) in the control as compared to the CEP55 shRNA cell, these regions were depicted green (knock-down$_{gain}$ < control$_{gain}$ | knock-down$_{loss}$ > control$_{loss}$). Green: regions closer to baseline in knock-down as compared to control. Red: regions further away from baseline in knock-down as compared to control. Blue: un-changed within 2*var(MedianRatio). For example: chromosomal region 1q is lost in control but not in the knockdown cell line. Therefore, 1q is depicted as a green positive value.

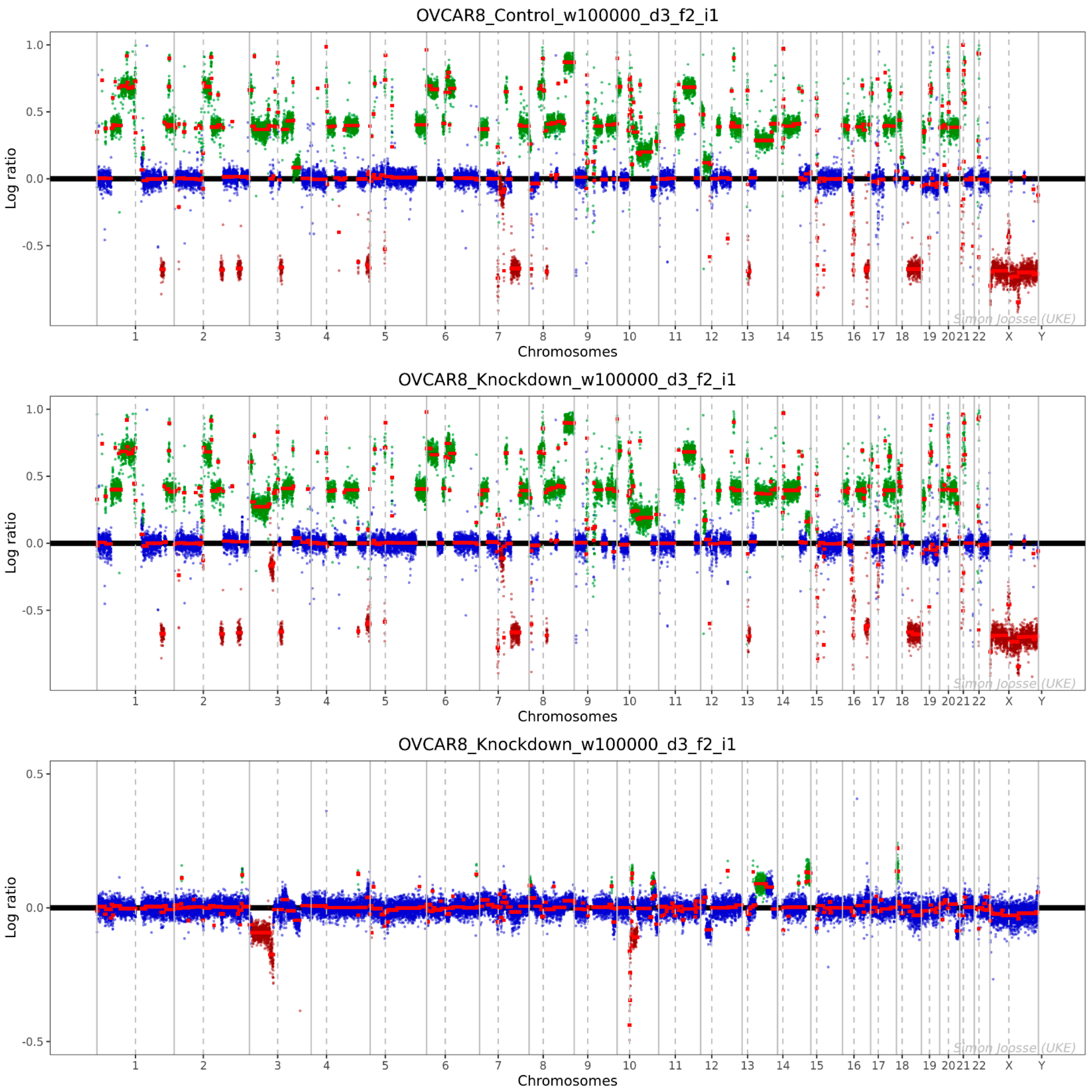

Figure S4. Copy number alterations (CNAs) in OVCAR-8 cells. The upper panel depicts the CNAs of the control OVCAR-8 cells, whereas the middle panel depicts the CNAs of the CEP55 knock-down cells. The chromosomes are depicted on the x-axis, with the solid lines signifying the chromosomal borders and the dotted lines the centromeres. The copy number levels are estimated based on the measure log ratio and copy number gains are depicted in green (positive log ratio), losses in red (negative log ratio), and no change in blue (log ratio around 0). The bottom panel depicts the difference between the control and knockdown cell lines. Log ratios corresponding to the same genomic loci were subtracted (knock-down – control). More extreme copy number alterations in the CEP55 knock-down cell lines as compared to the control cell lines (knock-down_{gain} > control_{gain} | knock-down_{loss} < control_{loss}), are described as "further away from baseline", and are depicted as red shaded regions.

However, if the copy number alterations were more extreme (i.e., further away from baseline) in the control as compared to the CEP55 shRNA cell, these regions were depicted green ($\text{knock-down}_{gain} < \text{control}_{gain}$ | $\text{knock-down}_{loss} > \text{control}_{loss}$). For example: chromosomal region 3cen is lost in the knock-down, but not in the control cell line. Therefore, 13q is depicted as a green positive value. Green: regions closer to baseline in knock-down as compared to control. Red: regions further away from baseline in knock-down as compared to control. Blue: un-changed within 2*var(MedianRatio).

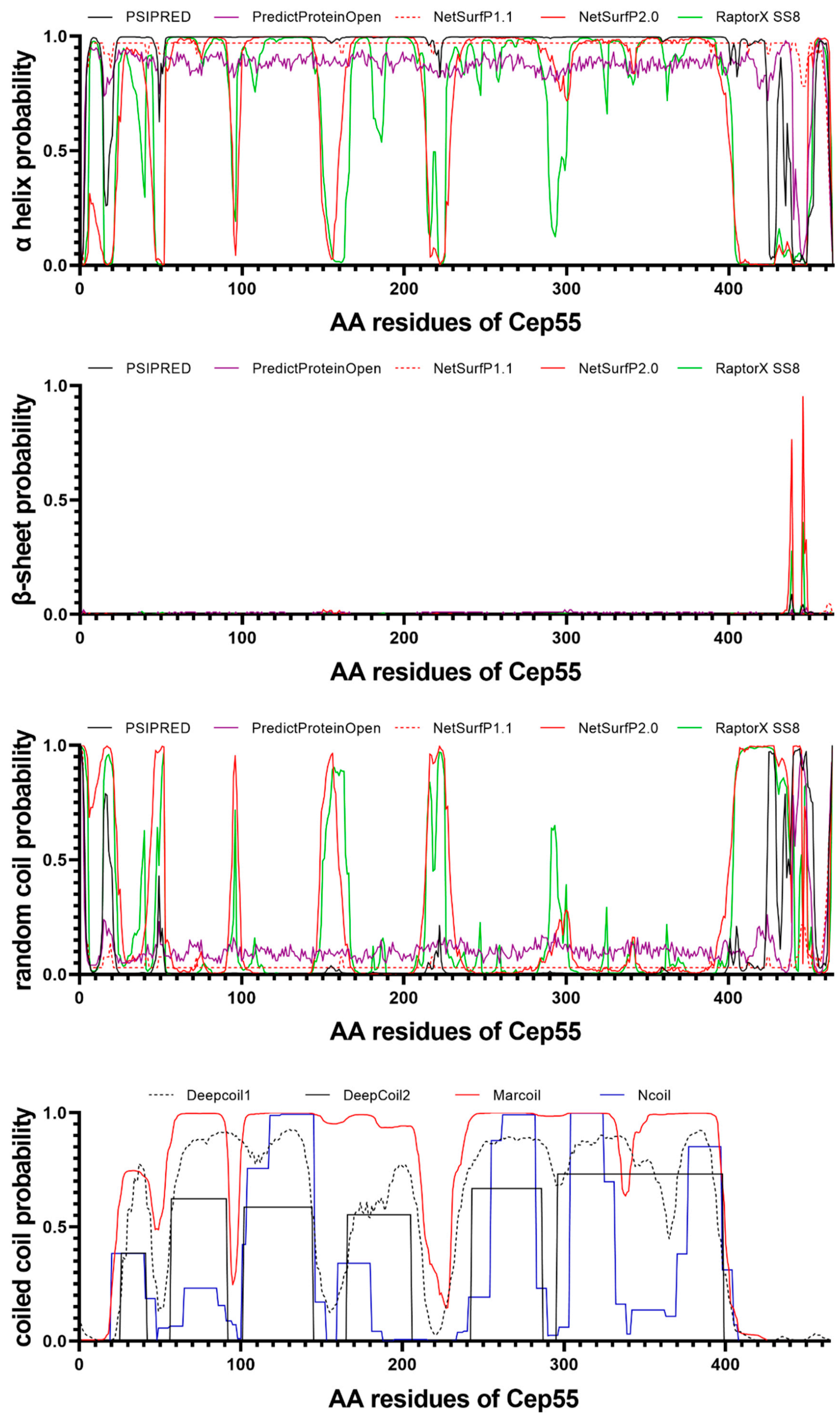

Figure S5. Predictions of the CEP55 secondary structure. Programs indicated in the Figure were used to predict α helices, β sheets, random coiled and coiled-coiled domains.

CEP55^{FL}

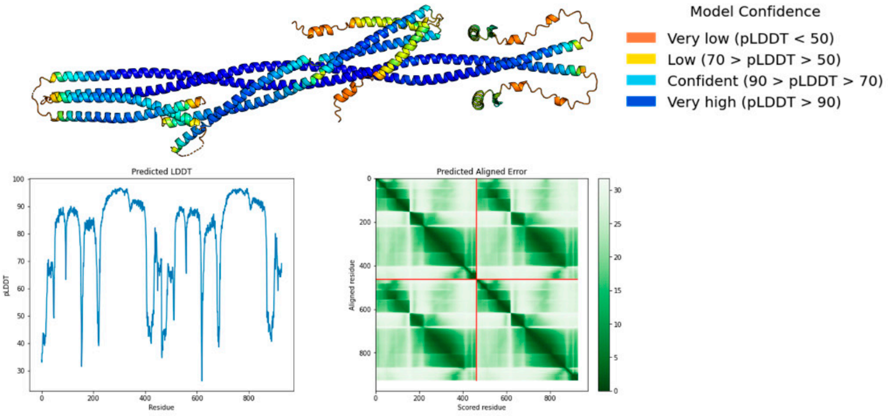

CEP55$^{59\text{-}464}$

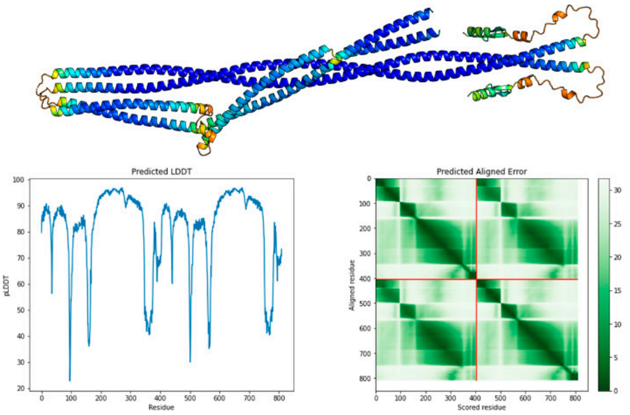

CEP55$^{1\text{-}428}$

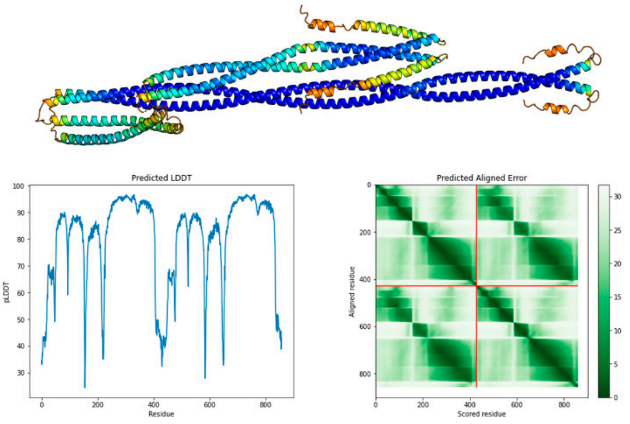

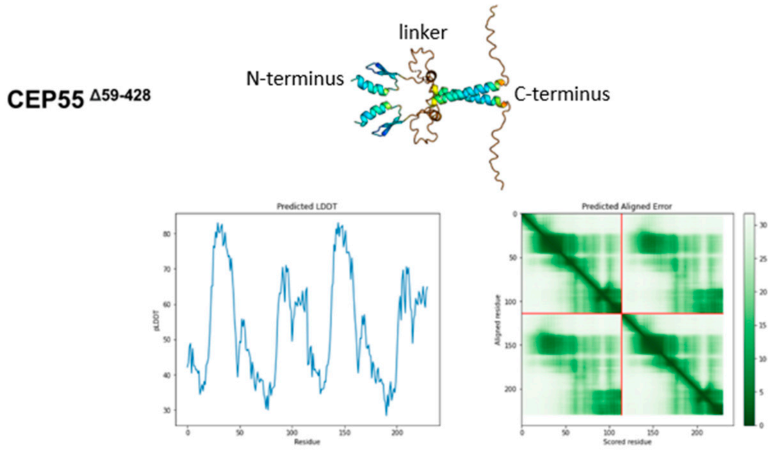

Figure S6. Prediction of CEP55 and CEP55 mutants by AlphaFold. Overview of the AlphaFold 2-predicted Cep55-dimer constructs, coloured in their per-residue confidence. In addition to the cartoon, the first graph also illustrates the per-residue confidence (pLDDT), noteworthy to mention that lower confidence bands are heavily correlated with disorder. The second graph indicates the Predicted Aligned Error (PAE), which is necessary to assess confidence in the domain packing and large-scale topology of the protein.

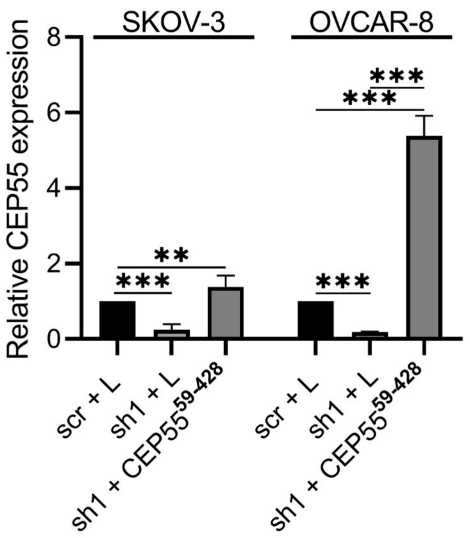

Figure S7. Re-expression of a CEP55 mutant with deleted MT-binding domain in sh1 cells. Expression of CEP55 mRNA was assessed by real time PCR. **p<0.001, ***p<0.0001.

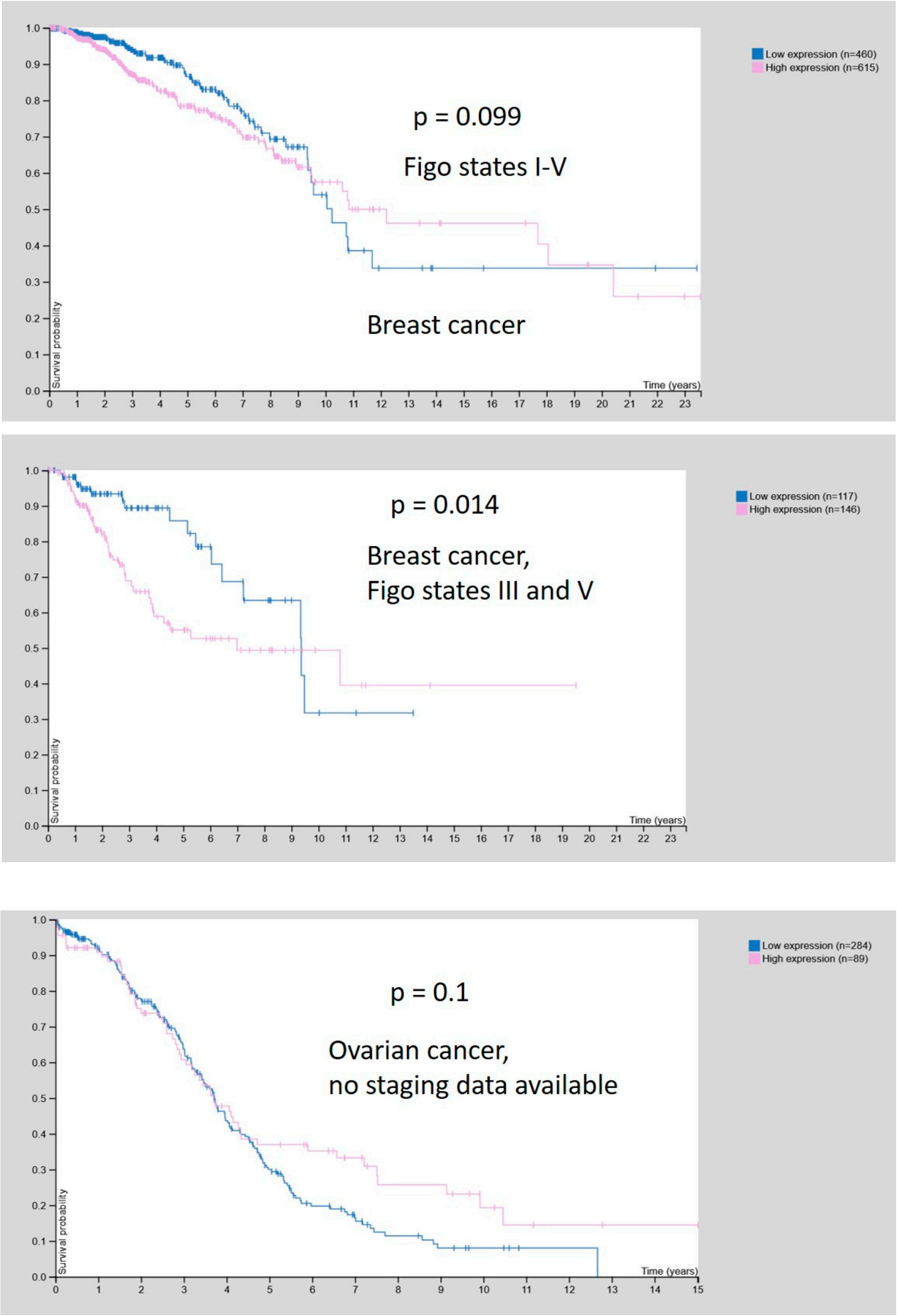

Figure S8. Kaplan Meier analysis of survival probability of breast and ovarian cancer patients and CEP55 expression. The data were extracted from THE HUMAN PROTEIN ATLAS: https://www.proteinatlas.org/ENSG00000138180-CEP55/pathology/breast+cancer; https://www.proteinatlas.org/ENSG00000138180-CEP55/pathology/ovarian+cancer#ihc, and represent mRNA data from the TCGA database
